# A double-network fish gelatin/sodium alginate composite hydrogel as a carrier for the sustained release of a soy-derived osteogenic peptide

**DOI:** 10.3389/fnut.2025.1733968

**Published:** 2025-12-15

**Authors:** Jinpeng Gong, Boya Liu, Tao Huang, Lu Wei, Yupeng Ma, Hao Chen, Jing Gan, Junbo Ge

**Affiliations:** 1 Department of Trauma Orthopedics, Yantaishan Hospital Affiliated to Binzhou Medical University, Yantai, China; 2 College of Food Science and Nutritional Engineering, China Agricultural University, Beijing, China; 3 Marine College, Shandong University, Weihai, Shandong, China; 4 College of Life Science, Yantai University, Yantai, Shandong, China

**Keywords:** composite hydrogel, soy peptide, osteoporosis, targeted delivery, carrier

## Abstract

Osteoporosis, a prevalent metabolic bone disease, poses a significant challenge for bone repair in aged populations. Soy-derived osteogenic peptide (SOP) holds promise but its clinical translation is hampered by rapid enzymatic degradation and poor oral bioavailability, representing a major challenge that necessitates the development of a protective delivery system. To address this, we developed a novel composite hydrogel based on fish gelatin and sodium alginate (SA/FG) as an oral delivery vehicle to overcome these limitations. Systematic characterization revealed that the SA/FG formulation offers distinct advantages: the incorporation of an optimal amount of SA significantly enhanced the mechanical strength and stability of the hydrogel, addressing the common weakness of single-network gelatin hydrogels, while maintaining a high water content (~90%) and a superior rehydration capacity (83.65% in distilled water). In a glucocorticoid-induced zebrafish osteoporosis model, the SOP-loaded SA/FG hydrogel significantly increased skull bone mass by 120.17% and improved larval swimming behavior, markedly outperforming the free SOP group. This study presents the first report of an SA/FG composite hydrogel for effective oral delivery of an osteogenic peptide. The system demonstrates great potential as a functional food or supplement for preventing and treating osteoporosis, offering an innovative strategy to enhance peptide stability and bioavailability through a biocompatible, double-network carrier.

## 1 Introduction

Osteoporosis and related bone injuries have emerged as a critical global public health challenge, with their prevalence and impact exacerbated by rapidly aging populations (1–3). This systemic skeletal disorder is characterized by reduced bone mass, microarchitectural deterioration of bone tissue, and an increased risk of fragility fractures, primarily resulting from an imbalance between osteoclastic bone resorption and osteoblastic bone formation (4, 5). The treatment and repair of osteoporotic bone defects thus represent a focal point of societal concern, holding significant implications for addressing aging-related challenges, alleviating healthcare burdens, and improving the quality of life for affected individuals (6, 7).

Among various strategies to promote bone repair, bioactive peptides have become the preferred osteogenic functional factors due to their high absorbability, ability to regulate diverse physiological activities, favorable stability, and excellent solubility (8–10). Compared to traditional small-molecule chemical drugs or large-molecular-weight protein growth factors, peptides offer distinct advantages including moderate molecular weight, low immunogenicity, specific bioactivity, and ease of synthesis and modification (11). They can mimic functional domains of natural proteins to effectively modulate cellular behaviors such as proliferation, differentiation, and mineralization of osteoblasts (12–14). Recent studies have identified specific osteogenic peptides from various food sources, underscoring their potential for bone health. For example, low molecular weight collagen peptides from bovine bone, phosphopeptides from egg yolk, and cyclolinopeptides from flaxseed have all demonstrated significant osteogenic effects in recent investigations (15–17). Notably, Wang et al. identified a soy peptide (SOP, amino acid sequence: VVELLKAFEEKF) from fermented soybean milk that exhibits osteogenic activity by promoting osteoblast differentiation and mineralization, demonstrating anti-osteoporotic potential in *in vivo* models (18, 19).

Despite their promise, a major technical challenge impedes the clinical application of food-derived bioactive peptides like SOP: their susceptibility to enzymatic degradation, rapid diffusion and clearance *in vivo*, and lack of targeting specificity, collectively resulting in low systemic bioavailability (20, 21). This limitation underscores the critical need for an appropriate delivery system capable of protecting the bioactive peptide, facilitating its targeted delivery to bone tissue, and enabling sustained release to maintain long-term therapeutic efficacy.

To address this delivery challenge, biopolymer-based hydrogels have been extensively investigated. Fish gelatin (FG), a natural polymer derived from fishery by-products, exhibits excellent biocompatibility, biodegradability, and low immunogenicity (22, 23). Its molecular structure is rich in Arg-Gly-Asp (RGD) sequences, which are known to effectively promote the adhesion, spreading, and proliferation of osteoblasts, thereby providing a favorable cellular microenvironment for bone repair (9, 24). However, single-network FG hydrogels often suffer from insufficient mechanical strength, a tendency to swell excessively under physiological conditions, and overly rapid degradation rates, which limit their practical application (25–27). A promising strategy to overcome these limitations is to blend FG with natural polysaccharides to form composite or double-network hydrogels (28). Sodium alginate (SA), a natural anionic polysaccharide extracted from brown algae, is widely used in biomedical applications due to its unique ion-crosslinking properties (e.g., rapid gelation in the presence of Ca^2+^) and good biocompatibility (29, 30). Previous studies, including those on protein-polysaccharide binary systems, have suggested that the combination of SA and FG can produce significant synergistic effects, improving the mechanical robustness, flexibility, and stability of the resulting composite hydrogels (31–33).

Therefore, the objective of this study was to prepare and characterize SA/FG composite hydrogels, evaluate their performance as carriers for soy peptides, and validate their osteogenic activity through *in vitro* cell experiments. This work provides a theoretical foundation and practical basis for developing a new class of high-efficiency, bioactive peptide-based delivery systems for the prevention and treatment of osteoporosis.

## 2 Materials and methods

### 2.1 Materials and equipments

FG with model 250BL-60 was obtained from Cargill Asia Pacific Food Systems Co., Ltd. (Beijing, China); analytical-grade sodium chloride was purchased from Tianjin Ruijinte Chemical Co., Ltd. (Tianjin, China); analytical-grade silicone oil was purchased from Fine Chemical Plant, Laiyang Economic and Technological Develop-ment Zone (Laiyang, China); food-grade sodium alginate was purchased from Zhengzhou Shiquan Shimei Trading Co., Ltd. (Zhengzhou, China). SOP (soybean-derived osteogenic peptide, sequence: VVELLKAFEEKF; molecular weight 1,475 Da) was synthesized by solid-phase peptide synthesis (SPPS) and supplied at a purity of ≥95% (Nanjing Peptide Biotechnology Co., Ltd., Nanjing, China). All other chemicals are of analytical grade and do not require further purification.

The following instruments were used in this study: a YP601N type electronic balance (Accuracy Class III; Shanghai Precision & Scientific Instrument Co., Ltd.), a CP224C type electronic balance (Accuracy Class I; Ohaus Instruments (Shanghai) Co., Ltd.), an RH-KT/C magnetic stirrer (IKA Works Guangzhou), and an EMS-12 magnetic stirrer (Tianjin Ouno Instrument Co., Ltd.). Additionally, a B-260 constant-temperature water bath (Shanghai Yarong Biochemical Instrument Factory), a DHG-9140A electric thermostatic drying oven (Shanghai Jinghong Laboratory Equipment Co., Ltd.), an NR110 precision colorimeter (Shenzhen Threenh Technology Co., Ltd.), and a Hakke Mars 3 rheometer (Hakke, Germany) were employed.

### 2.2 Preparation of FG single-network hydrogels

Preliminary trials indicated poor gelation capability of FG below 5% concentration, with complete failure to form cohesive hydrogels. Consequently, experimental concentrations were established at 6, 7, 8, 9, and 10% (w/v). For each concentration, 3.0, 3.5, 4.0, 4.5, or 5.0 g of FG powder was weighed into separate 100 mL beakers. After adding 50 mL distilled water, solutions were magnetically stirred for 1 h at 25 °C to ensure complete dissolution, followed by hydration at 4 °C for 24 h. Hydrated samples were then incubated in a 60 °C water bath for 30 min. Upon full liquefaction, 50 g aliquots were transferred to Petri dishes, equilibrated at room temperature for 20 min, and finally gelled at 4 °C for 24 h.

### 2.3 Preparation of SA/FG composite hydrogels

Composite hydrogels were prepared by combining 8% FG with varying SA concentrations (1, 1.5, 2, 2.5, 3% w/v). For each formulation, FG (4.0 g) and SA (0.5, 0.75, 1.0, 1.25, or 1.5 g, respectively) were co-dissolved in 50 mL distilled water within 100 mL beakers. After 1 h of magnetic stirring at 25 °C, solutions were hydrated at 4 °C for 24 h. Hydrated mixtures were then homogenized using a thermostated magnetic stirrer at 60 °C for 30 min, equilibrated at room temperature for 20 min, and finally gelled at 4 °C for 24 h prior to analysis.

Based on previous studies (18), SOP exhibits optimal osteogenic activity at micromolar concentrations. Therefore, SOP was incorporated into the SA/FG composite solution at a final concentration of 30 μM prior to the gelation process. The mixture was homogenized at 60 °C for 30 min, degassed for 20 min, and finally gelled at 4 °C for 24 h to obtain the SOP-loaded SA/FG composite hydrogels.

### 2.4 Characterization of hydrogels

#### 2.4.1 Measurement of color values

The color values of SA/FG composite hydrogels with different SA contents were measured using a precision spectrophotometer. The CIE Lab color parameters L*, a*, and b* were recorded, respectively (34). L* represents the lightness index, with L* = 0 indicating black and L* = 100 indicating white; a* represents red and green, where positive a* indicates red and negative a* indicates green; b* represents blue and yellow, where positive b* indicates yellow and negative b* indicates blue. Color values can be used to objectively evaluate the magnitude of color differences and their visual distinctions. The significance of the color difference value Δ*E* is generally shown in Table 1. Δ*E* can be calculated using the following formula.


$$\mathrm{\Delta E}={\left[{\left(L*-L\right)}^{2}+{\left(a*-a\right)}^{2}+{\left(b*-b\right)}^{2}\right]}^{\frac{1}{2}}$$

**Table 1:** General color values and their corresponding meanings.

Color values	Implications
1.6 < Δ*E* < 3.2	Cannot distinguish its color difference
3.2 < Δ*E* < 6.5	A few people can tell the difference in colors
6.5 < Δ*E* < 13	The color difference is very obvious
13 < Δ*E* < 25	Most belong to different colors
Δ*E* > 25	Different colors

L*, a*, and b* are the color parameter values of the hydrogel samples, while L, a, and b are the color parameter values of the control hydrogel (without SA).

#### 2.4.2 Determination of water content in hydrogels

The empty aluminum dish was weighed and recorded as m0. Approximately 10 g of hydrogel sample was transferred into the dish, with the total mass recorded as m1. The sample-containing dish was then oven-dried until constant weight was achieved, and the final mass was recorded as m2. The hydrogel water content was calculated us-ing the following formula:


$$\text{Water cotent}\%=\frac{m_{2}-m_{0}}{m_{1}-m_{0}}\times 100\%$$

#### 2.4.3 Determination of rehydration ratio in hydrogels

To each dried sample from the preceding procedure, sufficient volumes of distilled water, tap water, and normal saline (0.9% NaCl) were added. After equilibration, pre-weighed hydrated gauze (recorded as m_3_) received the rehydrated sample. Excess surface moisture was removed by blotting with filter paper until no dripping occurred. The combined mass of gauze and sample was recorded as m_4_. The rehydration ratio was calculated as follows:


$$\text{Rehydration ratio}\%=\frac{m_{4}-m_{3}}{m_{2}-m_{0}}\times 100\%$$

#### 2.4.4 Rheological characterization in hydrogels

Rheological characterization was performed using a P35TiL rotor with 35-mm parallel plates (1 mm gap) at 0.5% strain and 1 Hz frequency. After homogenization, a 1 mL bubble-free aliquot from the lower phase of the solution was rapidly transferred via micropipette onto the rheometer stage preheated to 60 °C. Sample edges were sealed with silicone oil to prevent moisture evaporation, followed by 5-min thermal equilibration. Temperature sweeps were conducted at 5 °C/min: cooling from 60 °C to 10 °C, holding at 10 °C for 10 min, then heating back to 60 °C. Storage modulus (G′) and loss modulus (G″) were continuously monitored throughout thermal cycles. Subsequent frequency sweeps (0.1–100 Hz) at isothermal 10 °C recorded the evolution of both moduli (35).

### 2.5 Assessment of antiosteoporosis effects in a zebrafish model of GIOP

#### 2.5.1 Zebrafish husbandry and maintenance

Adult wild-type AB zebrafish were obtained from the China Zebrafish Resource Center (Beijing, China). Adults were maintained at 28.5 °C in a recirculating aquaculture system under a 14 h light/10 h dark cycle. Embryos and larvae were reared in fish water containing 5.0 mM NaCl, 0.17 mM KCl, 0.33 mM CaCl₂, and 0.33 mM MgSO_4_. The animal study protocol was approved by the Animal Care and Use Committee (ACUC) of Yantai University, protocol number YT 20250078. The study adhered to the guidelines set by the committee.

#### 2.5.2 Establishment of GIOP zebrafish model and experimental design

The zebrafish, a vertebrate species, exhibits strong physiological conservation in skeletal biology with mammals, making it an ideal model system for studying osteoporosis treatment (5, 36, 37). In this study, a glucocorticoid-induced osteoporosis (GIOP) zebrafish model was established according to the Barrett method (38). Normally developing 72 h post-fertilization (hpf) embryos were selected and continuously treated for 4 days in fish water containing 25 μM prednisolone along with either SOP or alendronate (ALN), with daily renewal of the medicated solution. Larvae at 7 hpf were subsequently collected for further experiments. The study comprised six experimental groups: 0.1% DMSO (vehicle), 25 μM prednisolone (model), coincubated SOP (30 μM), 0.308 μM ALN (positive control), SA/FG Composite Hydrogels (composite carrier), and SOP-loaded SA/FG Composite Hydrogels (SOP-loaded composite carrier).

#### 2.5.3 Calcein staining and behavioral analysis

Calcein, a fluorescent dye that binds specifically to calcified bone matrix, was used to assess bone mineral density in GIOP zebrafish by measuring skull fluorescence intensity (39). After treatment, 7 dpf larvae were stained for 5 min in fish water containing 0.2% (w/v) calcein, rinsed three times with clean water, anesthetized with 0.016% (w/v) MS-222, and mounted on concave slides using 3% (w/v) methylcellulose (Aladdin, Shanghai, China). Images were acquired using a fluorescence microscope (Leica DMi8, Wetzlar, Germany), with all procedures performed under light-protected conditions. The relative fluorescence intensity (RFI) of the skull bone mass was quantified using ImageJ software. Behavioral analysis was conducted in 96-well plates maintained at 27.5 ± 1 °C between 9: 00 a.m. and 12:00 p.m. Spontaneous embryonic movements were calculated based on tail coil frequency recorded over a 10-min period using a zebrafish behavior tracking system (Danio Vision, Noldus, Wageningen, Netherlands).

### 2.6 Statistical analysis

All data are presented as the mean ± standard error of the mean (SEMs) from three to six independent experiments. Data were analyzed using one-way analysis of variance (ANOVA) with GraphPad Prism 5.0 (GraphPad Software, San Diego, CA, USA) and Excel (Microsoft, San Francisco, CA, USA). A *p*-value < 0.05 was considered statistically significant.

## 3 Results and discussion

### 3.1 Sensory characteristics of SA/FG composite hydrogels

**Figure 1:**
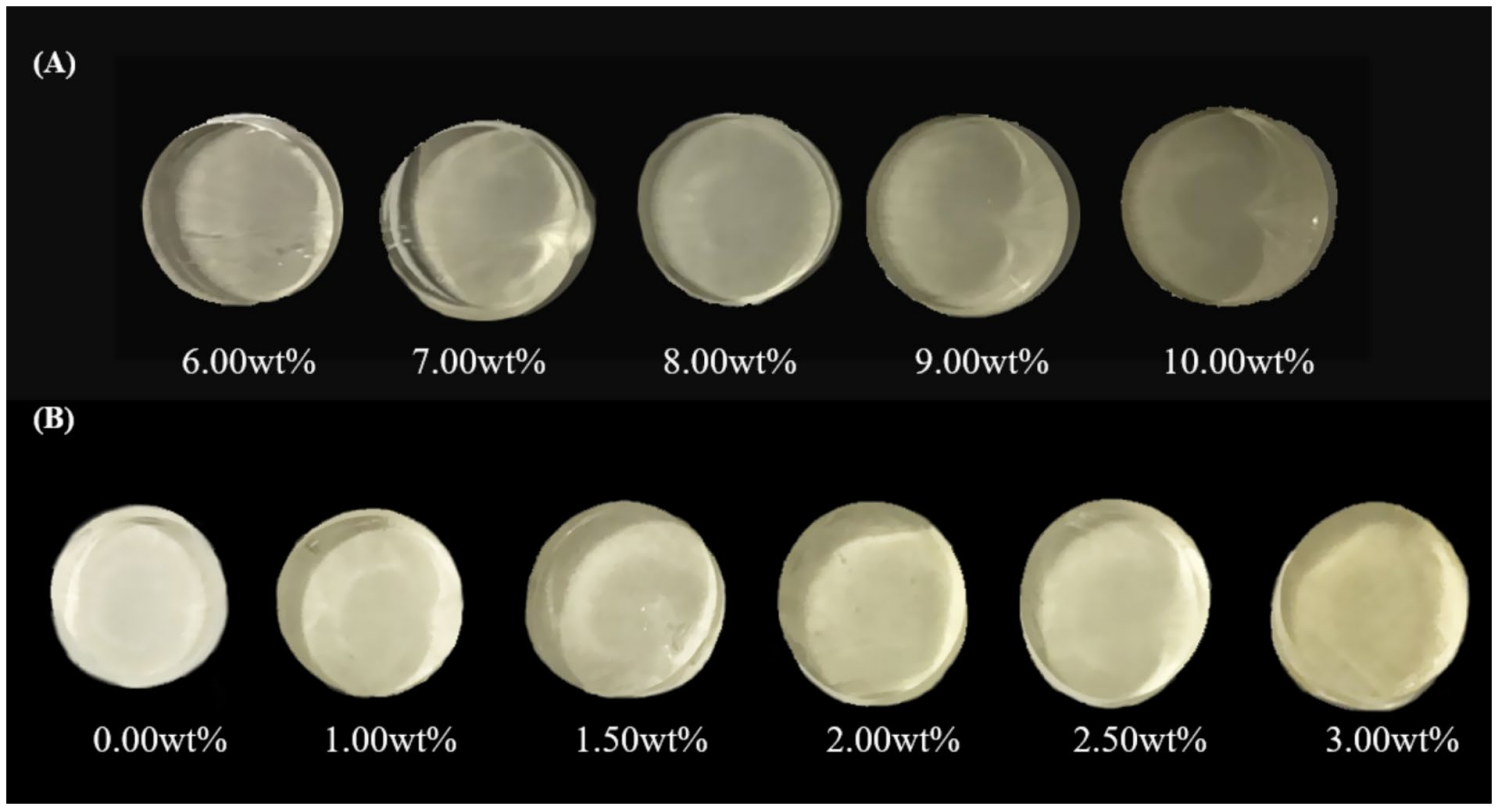
Photographs of single hydrogels with different FG contents **(A)**; photographs of the SA/FG composite hydrogels with different SA contents, (8% FG) **(B)**.

**Figure 2:**
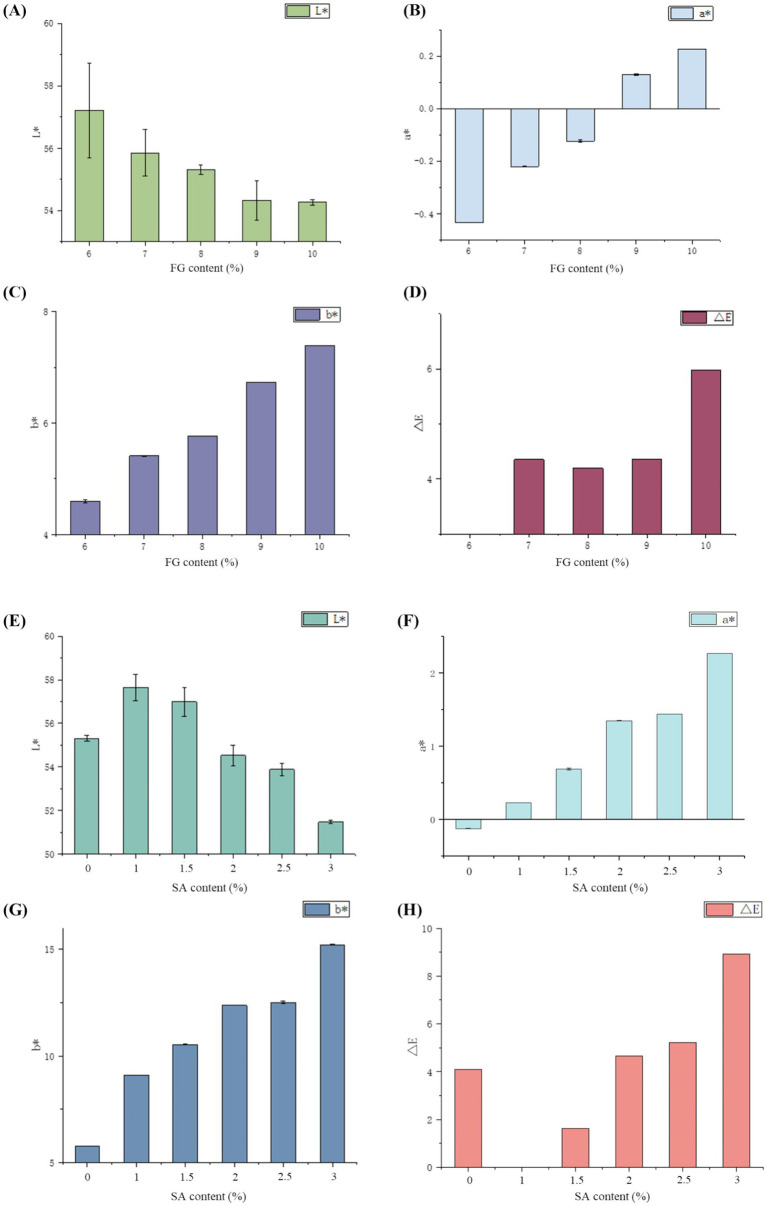
Effects of different FG concentrations on the color of single hydrogels **(A)** L*, **(B)** a*, **(C)** b*, and **(D)** Δ*E*; effects of SA content on the color of SA/FG composite hydrogels **(E)** L*, **(F)** a*, **(G)** b*, and **(H)** Δ*E*, (8% FG).

Photographs of FG single hydrogels at varying mass concentrations and SA/FG composite hydrogels as a function of SA content are shown in Figure 1. The changes in FG color at different mass concentrations and the color of the composite hydrogel as the SA content varies are shown in Figure 2. It can be observed that within the concentration range of 6–10%, the Δ*E* values were all below 6.5, indicating that color differences were undetectable to the naked eye. As shown in the figure, the L* values gradually decreased from approximately 57–54 with increasing concentration, while both a* and b* values increased. Research suggests that the light transmittance of hydrogels reflects their turbidity level. The transparency index can indicate the number of aggregated particles and the size of voids within the hydrogel, indirectly reflecting the degree of cross-linking and aggregation in the hydrogel network structure. Therefore, a correlation exists between the transmittance and hydrogel strength. Generally, hydrogels with higher turbidity exhibit greater hydrogel strength (40). Thus, the decrease in lightness with increasing concentration may be attributed to the rise in FG concentration, which raised the number of hydrogel aggregate particles, enhanced cross-linking and aggregation in the network structure, higher turbidity, lower light transmittance, and reduced light reflection from a white background—resulting in a decline in the lightness index.

When the SA content reached 3%, the Δ*E* of the composite hydrogels reached 8.9275, indicating a discernible color difference while the hue remained unchanged, specifically yellow. After adding SA, the composite hydrogels exhibited an initial increase followed by a decrease in L*, while both a* and b* progressively increased. This indicated that introducing low-concentration SA enhanced the lightness of the composite hydrogels, but exceeding a critical addition threshold reduced its lightness. The incorporation of an appropriate amount of SA reduced the degree of cross-linking in the composite hydrogels, creating intermolecular voids that facilitated light transmission and reflection, thereby enhancing the lightness index of the composite hydrogels. Kazemi-Taskooh found that polysaccharides have a space-occupying effect on protein hydrogels (41). Therefore, as the content of SA increased, many voids may have been occupied by SA, which was not conducive to light transmission and reduced the brightness index of the composite hydrogels.

Although increasing SA concentration darkens the hydrogels and alters its color characteristics, the hydrogels retains visual uniformity, thus maintaining satisfactory appearance.

### 3.2 The water content of hydrogels

**Figure 3:**
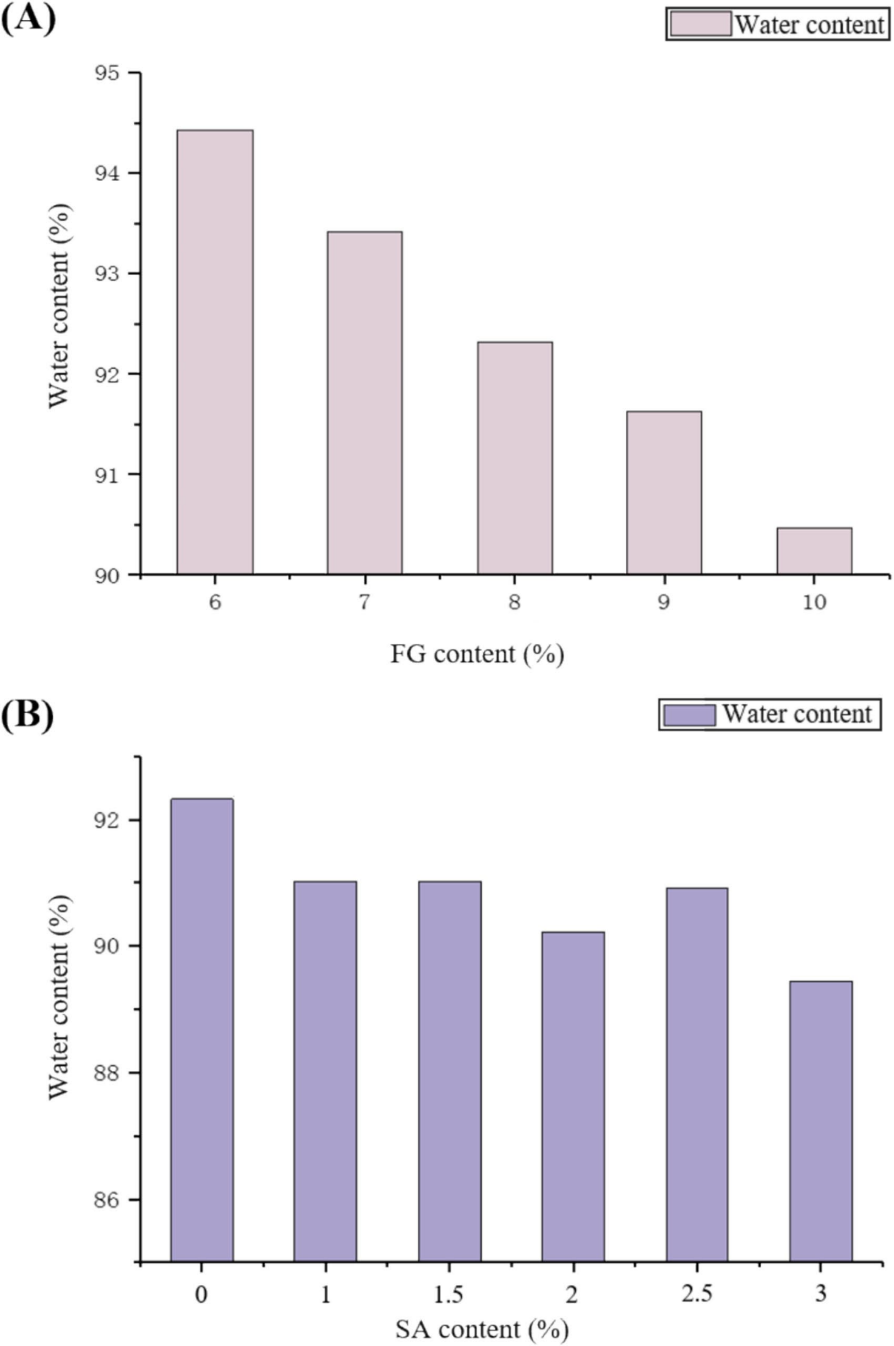
Water content of FG single hydrogels and SA/FG composite hydrogels at varying concentrations: **(A)** FG single hydrogels and **(B)** SA/FG composite hydrogels with 8% FG.

The water content of FG (A) and SA/FG composite hydrogels (8%FG) (B) at varying concentrations is shown in Figure 3. For the single-network FG hydrogels (Figure 3A), the water content decreased from 94.43 to 90.47% as the FG concentration increased from 6 to 10%. This inverse relationship can be attributed to the increased probability of intermolecular bonding and the formation of a more densely cross-linked network at higher biopolymer concentrations (42). The resulting compact structure inherently possesses a reduced capacity to entrap and hold water molecules, thereby leading to a lower overall water content.

The experiments revealed a gradual but marginal decrease in water content with SA addition. The water content of SA/FG composite hydrogels still ranged from 91.02 to 89.45%. All composite hydrogels maintained desirable water retention capacity. This likely stemmed from enhanced intermolecular interactions between FG and SA, which reorganized the network into a more finely structured and less porous matrix. While this compacted structure restricted water mobility and improved dehydration resistance, it also altered the pore architecture in a way that, at optimal SA concentrations, facilitated capillary action and water infiltration during rehydration. Consequently, while the water retention capacity slightly declined with increasing SA concentration. Compared with FG without SA, the composite hydrogels maintained substantial water retention capacity, effectively retaining sufficient water molecules. Crucially, this modulation in microstructure, which is responsible for the marginal reduction in water content, is directly linked to the formation of a more robust double-network structure, contributing to the enhanced mechanical stability observed in our rheological studies (43).

### 3.3 The rehydration ratio of hydrogels

**Figure 4:**
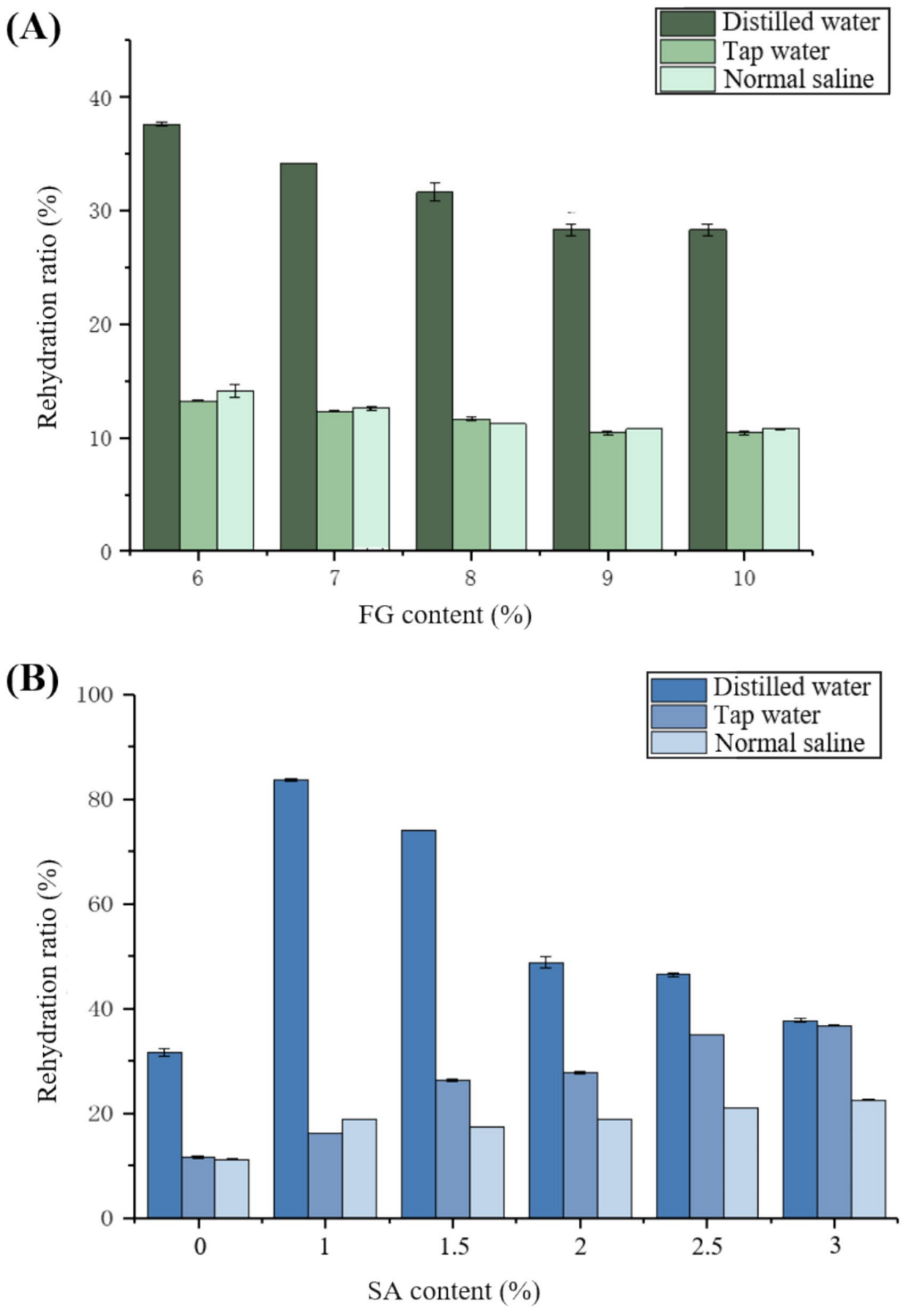
Rehydration ratio at varying concentrations in different solutions (distilled water, tap water, normal saline): **(A)** FG single hydrogels and **(B)** SA/FG composite hydrogels with 8% FG.

Figure 4 presents the effects of distilled water, tap water, and normal saline (0.9% NaCl) on the rehydration ratio of FG(A), SA/FG composite hydrogels (8% FG) (B) at varying concentrations. For the single-network FG hydrogels (Figure 4A), the rehydration ratio generally followed the order: distilled water > normal saline > tap water across most concentrations, with a maximum value of 37.61% achieved in distilled water. This trend can be explained by the absence of competing ions in distilled water, which allows for unimpeded water uptake by the hydrogel’s hydrophilic groups. In contrast, the presence of ions in tap water and normal saline (0.9% NaCl) can suppress the electrostatic repulsion between polymer chains and promote chain aggregation through a “charge screening effect,” leading to a more collapsed network structure with reduced water absorption capacity (44, 45). The rehydration ratio decreased with increasing FG concentration, as higher polymer content leads to a denser network with fewer and smaller pores available for water penetration and storage.

A remarkable enhancement in rehydration capacity was observed for the SA/FG composite hydrogels (Figure 4B). The incorporation of SA dramatically increased the rehydration ratio in distilled water, elevating it from 37.61% (pure FG) to 83.65%. This significant improvement is attributed to the introduction of SA, which, at optimal concentrations, interferes with the dense packing of FG chains, thereby creating additional intermolecular voids and a more open network structure that facilitates extensive water infiltration (46). However, beyond an optimal SA concentration (1%), the rehydration ratio in distilled water began to decline, suggesting that an excess of SA may fill these beneficial voids, resulting in a overly compact structure that hinders water penetration.

Notably, the composite hydrogels also exhibited an improved rehydration capacity in ionic media (tap water and normal saline) compared to the pure FG hydrogel, and this capacity increased with SA content. This can be rationalized by the high density of carboxylate groups (–COO) on the SA chains. These anionic groups can bind water molecules strongly through hydration effects. Even in the presence of ions, this intense hydration shell helps to maintain a more swollen state and resist the network collapse typically induced by charge screening, thereby preserving a higher rehydration capacity (41). This behavior underscores the advantage of the double-network structure, which not only enhances water uptake in pure water by creating a more open architecture but also improves water retention in physiological ionic environments by introducing highly hydrophilic components.

### 3.4 The rheological properties of hydrogels

**Figure 5:**
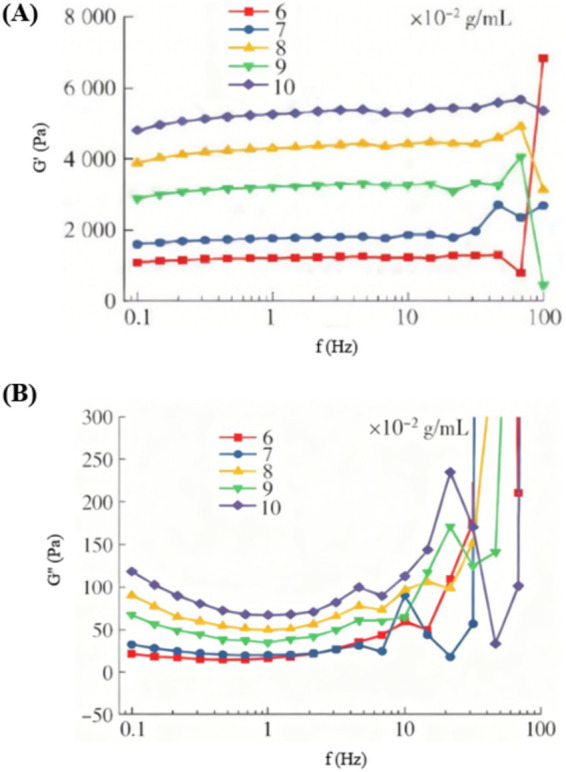
Frequency dependence of **(A)** G′ and **(B)** G″ for FG single hydrogels at varying concentrations (10 °C).

Figure 5 presents the frequency-dependent variations in storage modulus (G’) and loss modulus (G”) for FG at varying concentrations, measured at 10 °C. All pure gelatin hydrogels exhibited significantly higher G’ than G,” confirming their dominant elastic solid-like behavior. Both moduli progressively increased with rising gelatin concentration, indicating enhanced network integrity and gel strength at higher concentrations. The 0.10 g/mL formulation demonstrated the most robust textural properties, which is consistent with the sensory evaluation results. Both G′ and G″ moduli increased with frequency, affirming the viscoelastic character of the FG hydrogels. At approximately 85 Hz, G″ surged abruptly while G′ dropped sharply, converging at this point. This crossover signified reduced elasticity and enhanced viscous dominance, as heightened molecular energy under rapid deformation induced structural disintegration of the colloidal network.

**Figure 6:**
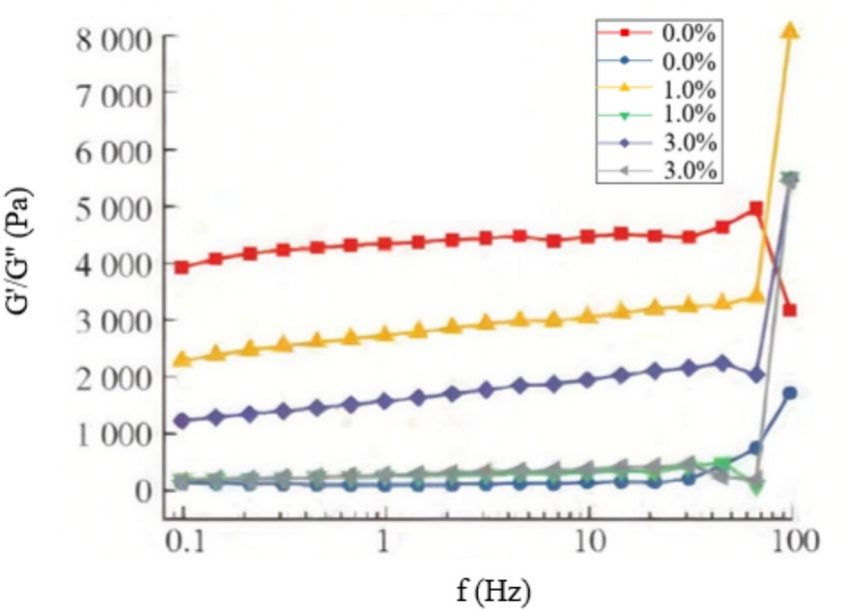
Frequency dependence of G′ and G″ for composite hydrogels with different SA concentrations (10 °C).

Figure 6 shows the frequency-dependent variations in G′ and G″ for the composite hydrogels with SA concentrations of 0.0, 1.0, and 3.0%, measured at 10 °C. The incorporation of SA modulated the mechanical properties, as evidenced by the relative changes in G′ and G″ (47). Although increasing SA concentration slightly reduced the absolute G′ values, it fundamentally altered the failure mechanism of the hydrogel. Crucially, G′ consistently remained higher than G″ across all systems (48), confirming the persistent gel-like elasticity. A key finding was observed at high frequencies (~75 Hz): while the single-network 0.08 g/mL FG hydrogel underwent structural failure, the SA/FG composite hydrogels exhibited a sharp increase in both moduli, with a particularly pronounced enhancement in G′. This demonstrates that the introduction of SA forms a reinforcing double-network structure that significantly improves the mechanical strength and stability of the hydrogel, effectively preventing catastrophic failure under high shear stress. This reinforcement effect is consistent with previous findings that the integration of SA into a FG matrix leads to the formation of a denser and more integrated network structure, thereby contributing to the enhanced mechanical integrity (49). Further frequency increases eventually amplified G″, signaling the onset of network failure, but at substantially higher stress levels compared to the pure FG hydrogel.

### 3.5 *In vivo* preventive effects of SOP-loaded composite hydrogels on osteoporosis

**Figure 7:**
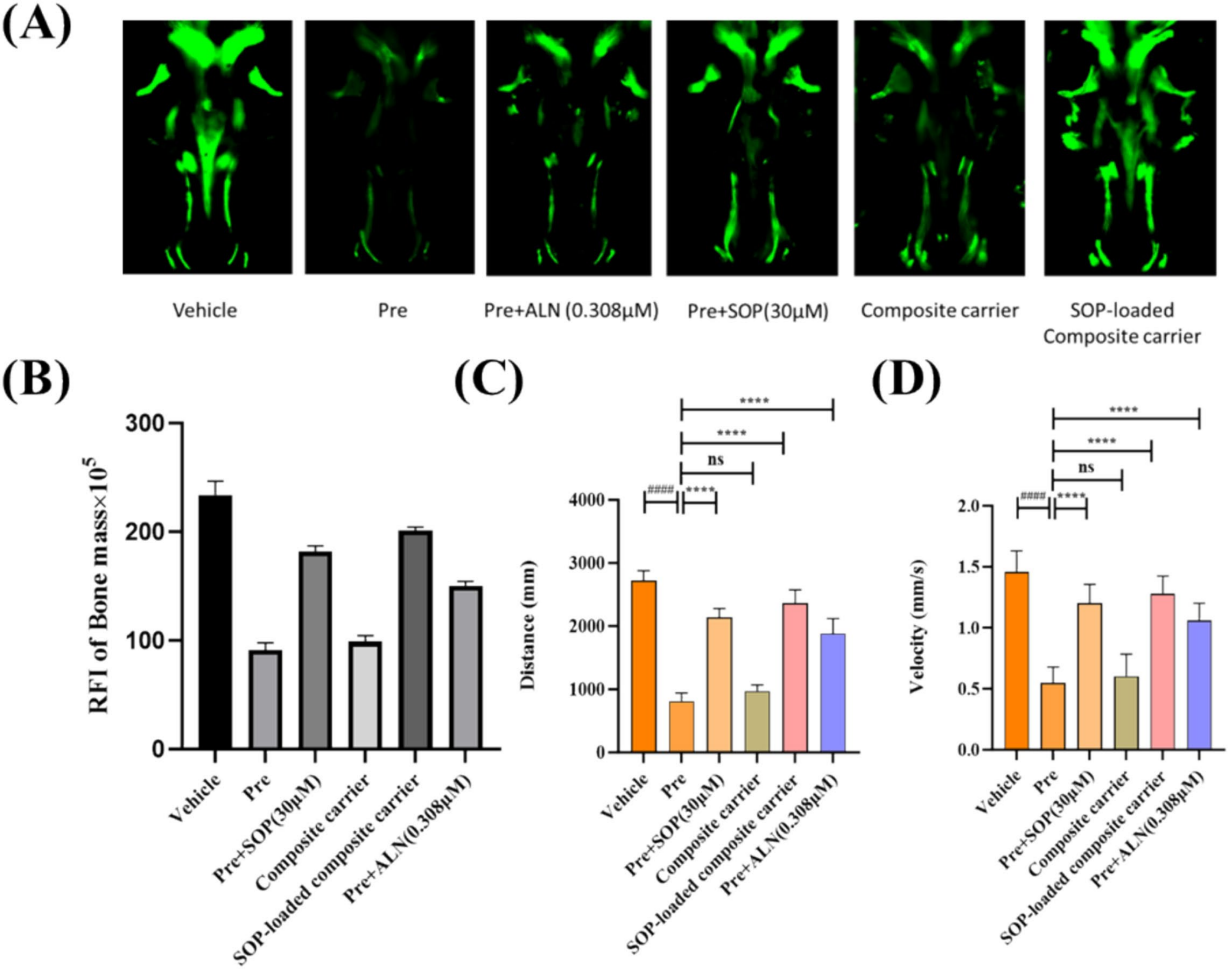
Effects of SOP-loaded composite hydrogels on osteoporosis in vivo preventive. **(A)** Illustrative fluorescence images of the zebrafish skull with varied samples at 7 dpf (a) 0.1% DMSO (vehicle), (b) 25 μM prednisolone (for osteoporosis), (c) 0.308 μM alendronate (as the positive control) or coadministered with SOP (30 μM) (d–f) for 96 h. **(B)** At 7 days postfertilization (dpf), the relative fluorescence intensity (RFI) of the zebrafish skull was quantified. **(C)** The distance travelled by zebrafish larvae at 96 hpf after being exposed to SOP. **(D)** The swimming speed of zebrafish larvae at 96 hpf after being exposed to SOP. *n* = 6–10. The data are presented as the mean ± SEM. Significant differences were observed as ##*p* < 0.01, ###*p* < 0.001, *p < 0.05, **p < 0.01, and ***p < 0.001 for comparisons vs. the vehicle, and vs. Pre, respectively, ns, not significant. (Pre, prednisolone; ALN, alendronate).

Wang et al. demonstrated that a SOP concentration of up to 30 μM is both safe and optimally effective for evaluating its impact in a prednisolone-treated zebrafish larval model (5). Figure 7A shows representative images of the zebrafish skull at 7 dpf, while Figure 7B presents the quantitative results of the relative fluorescence intensity (RFI), reflecting skull bone mass. Compared with the vehicle control group, the model group exhibited a significant reduction in skull fluorescence intensity by 60.93% (*p* < 0.001). In contrast, treatment with 0.308 μM ALN reversed this effect, increasing the fluorescence intensity by 64.66% compared to the model group (*p* < 0.001), confirming the successful establishment of the GIOP model. Treatment with SOP (30 μM) significantly increased the bone mass RFI by 98.71% compared to the model group (*p* < 0.01), demonstrating its ability to markedly reverse prednisolone-induced bone mineral density loss. However, the skull fluorescence intensity in the SOP group remained significantly lower than that in the control group, primarily attributed to SOP’s susceptibility to enzymatic degradation *in vivo*, which limits its stability and bioavailability. The composite carrier alone only increased the bone mass RFI by 8.34% compared to the model group, indicating that the gel itself had no significant therapeutic effect on bone density. Notably, the SOP-loaded composite hydrogel significantly increased the bone mass RFI by 120.17% compared to the model group, achieving efficacy comparable to the 0.308 μM ALN treatment. This confirms that the composite hydrogel provides excellent anti-osteoporotic efficacy through the sustained release of SOP, protecting its bioactivity and enabling long-lasting, controlled release, thus representing an ideal delivery vehicle.

Osteoporosis also affected zebrafish behavioral performance (50, 51). Analysis of locomotor capacity using a zebrafish behavior tracking system (Figures 7C,D) revealed that compared to the vehicle group, prednisolone significantly reduced the total distance travelled and average swimming speed of the zebrafish. These behavioral deficits were reversed by 0.308 μM ALN. Treatment with 30 μM SOP increased the total distance travelled in 10 min from 809.1 mm to 2143.0 mm and the average swimming speed from 0.55 mm/s to 1.20 mm/s (*p* < 0.05). The SOP-loaded composite hydrogel increased the total distance travelled in 10 min from 809.1 mm to 2236.1 mm and the average swimming speed from 0.55 mm/s to 1.28 mm/s (*p* < 0.05), indicating that the SOP-loaded SA/FG composite hydrogel partially ameliorated the behavioral deficits in GIOP zebrafish. In summary, the SOP-loaded composite hydrogel effectively reversed both bone loss and locomotor dysfunction in the GIOP zebrafish model.

## 4 Conclusion

In conclusion, this study successfully developed and systematically evaluated a novel composite hydrogel based on fish gelatin and sodium alginate (SA/FG) as an effective oral delivery carrier for a soy-derived osteogenic peptide (SOP). The incorporation of an optimal amount of SA was found to be critical, as it significantly enhanced the mechanical strength and stability of the hydrogel, as evidenced by rheological analysis, while maintaining a high water content (~90%) and superior rehydration capacity (up to 83.65% in distilled water). Most importantly, in a glucocorticoid-induced osteoporosis zebrafish model, the SOP-loaded SA/FG composite hydrogel demonstrated remarkable anti-osteoporotic efficacy, increasing skull bone mass by 120.17% and effectively reversing behavioral deficits, outperforming the free SOP. This study highlights the dual advantage of the SA/FG double-network system: it protects the bioactive peptide from rapid degradation and enables its sustained release, thereby significantly improving bioavailability and osteogenic activity. While the current findings are promising, future work will focus on elucidating the precise peptide release kinetics, the long-term biodegradation profile of the hydrogel, and its therapeutic efficacy in mammalian bone defect models to further advance its clinical translation.

## Data Availability

The raw data supporting the conclusions of this article will be made available by the authors, without undue reservation.
