# Supplementary material for: A double-network fish gelatin/sodium alginate composite hydrogel as a carrier for the sustained release of a soy-derived osteogenic peptide
Source: Front Nutr. 2025 Dec 15;12:1733968. doi: 10.3389/fnut.2025.1733968 (PMC12746747; doi:10.3389/fnut.2025.1733968)
Supplement: Supplementary file 1 [file Data_Sheet_1.pdf]

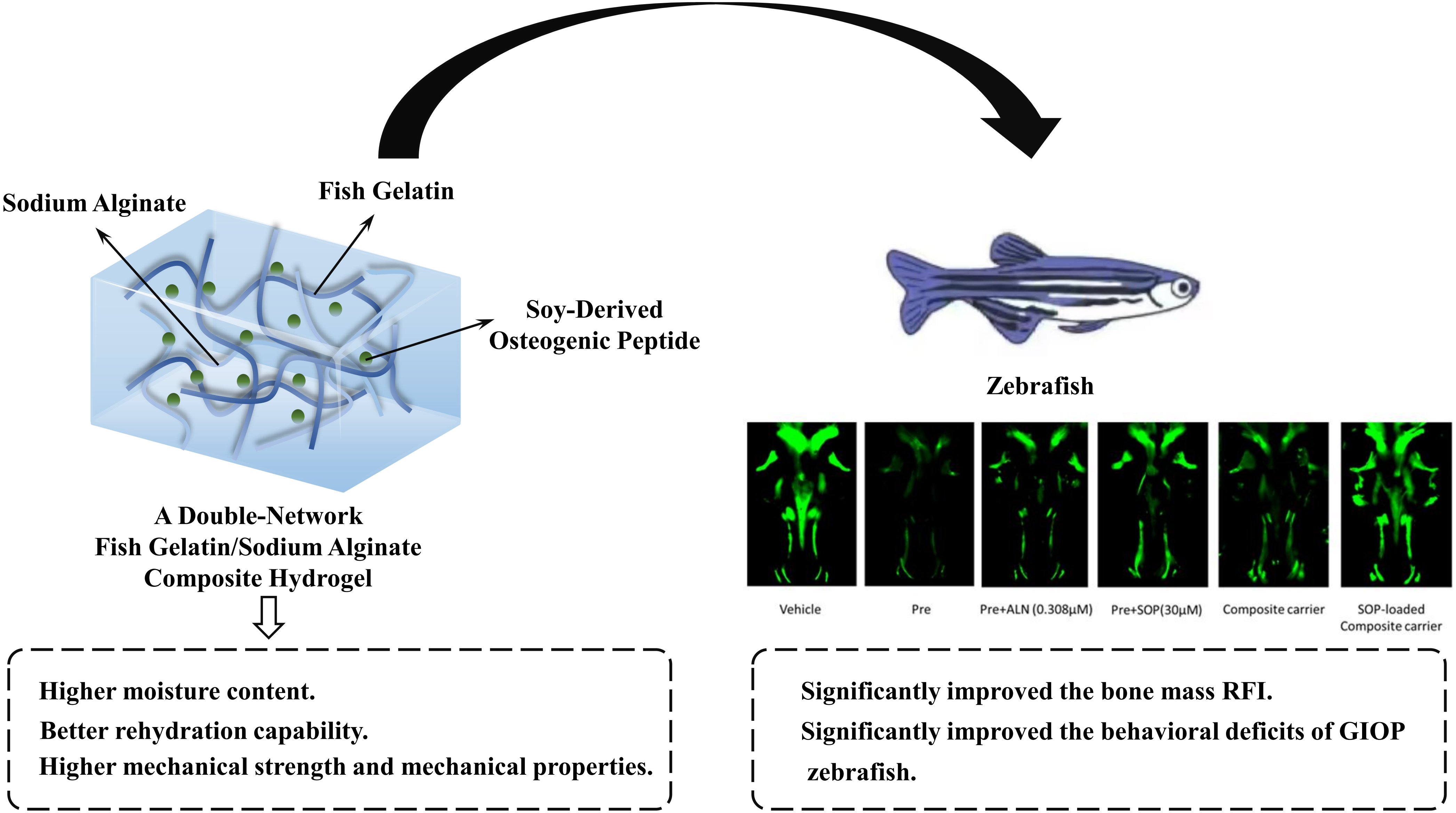
Sodium Alginate
Fish Gelatin
Soy-Derived
Osteogenic Peptide
A Double-Network
Fish Gelatin/Sodium Alginate
Composite Hydrogel
Higher moisture content.
Better rehydration capability.
Higher mechanical strength and mechanical properties.
Zebrafish
Vehicle
Pre
Pre+ALN (0.308μM)
Pre+SOP(30μM)
Composite carrier
SOP-loaded
Composite carrier
Significantly improved the bone mass RFI.
Significantly improved the behavioral deficits of GIOP
zebrafish.
